# Costs and cost-effectiveness of palliative care in residential aged care homes: a scoping review

**DOI:** 10.1093/ageing/afag217

**Published:** 2026-07-24

**Authors:** Xueqing Yang, Fengyi Liu, Margaret MacAndrew, Nicole White, Hannah Elizabeth Carter

**Affiliations:** Queensland University of Technology, Australian Centre for Health Services Innovation and Centre for Healthcare Transformation, School of Public Health and Social Work, Faculty of Health, 88 Musk Avenue, Kelvin Grove, Brisbane, Queensland 4059, Australia; Queensland University of Technology, Australian Centre for Health Services Innovation and Centre for Healthcare Transformation, School of Public Health and Social Work, Faculty of Health, 88 Musk Avenue, Kelvin Grove, Brisbane, Queensland 4059, Australia; Queensland University of Technology, Centre for Healthcare Transformation, School of Nursing, Faculty of Health, N Block, Victoria Park Road, Kelvin Grove, Brisbane, Queensland 4059, Australia; Queensland University of Technology, Australian Centre for Health Services Innovation and Centre for Healthcare Transformation, School of Public Health and Social Work, Faculty of Health, 88 Musk Avenue, Kelvin Grove, Brisbane, Queensland 4059, Australia; Queensland University of Technology, Australian Centre for Health Services Innovation and Centre for Healthcare Transformation, School of Public Health and Social Work, Faculty of Health, 88 Musk Avenue, Kelvin Grove, Brisbane, Queensland 4059, Australia

**Keywords:** palliative care, end-of-life care, cost-effectiveness, healthcare costs, nursing home, older people

## Abstract

**Background:**

Palliative care is increasingly recognised as a core component of healthcare delivery in residential aged care (RAC) homes, yet its economic implications remain unclear. Understanding costs and cost-effectiveness is essential for guiding resource allocation decisions in these settings.

**Objective:**

To map the evidence on the costs and cost-effectiveness of palliative care delivered in RAC homes, and to identify gaps in the evidence base.

**Methods:**

A scoping review was conducted in accordance with Joanna Briggs Institute guidelines and reported following the Preferred Reporting Items for Systematic Reviews and Meta-Analyses extension for Scoping Reviews checklist. Searches were conducted in the PubMed, SCOPUS, Embase, CINAHL and CENTRAL databases. Studies reporting on quantitative economic outcomes of palliative care in RAC settings were included. Findings were summarised using narrative synthesis.

**Results:**

Of the 3182 items identified, 15 studies were included. Three categories of economic outcomes were identified: care delivery costs (*n* = 3), healthcare system costs (*n* = 10) and cost-effectiveness outcomes (*n* = 2). Studies examining care delivery costs reported additional costs associated with palliative care. Among studies assessing healthcare system costs, six reported reduced costs, one reported increased costs and three reported mixed findings varying by time horizon and care continuity. Two full economic evaluations reported favourable cost-effectiveness findings compared with usual care, although the statistical significance of findings varied.

**Conclusion:**

Economic evidence on palliative care in RAC remains limited and methodologically heterogeneous, constraining robust conclusions regarding its economic value. Future research should prioritise rigorous economic evaluations that comprehensively capture healthcare and social care costs while incorporating patient-centred outcomes to better assess the value of palliative care in RAC homes.

## Key Points

- Economic evidence on palliative care in residential aged care remains limited and methodologically heterogeneous.
- Studies commonly report reduced healthcare costs associated with palliative care, although findings are inconsistent.
- High-quality economic evaluations are needed to better inform resource allocation and value-based care decisions.

## Background

The demand for palliative care is increasing globally as populations age and the prevalence of chronic, progressive and life-limiting illnesses continues to rise [1]. Palliative care is defined by the World Health Organisation as an approach that aims to improve the quality of life of patients and their families facing problems associated with life-limiting illness, by preventing and relieving physical, psychosocial and spiritual suffering [2].

Residential aged care (RAC) homes, also known as nursing homes and long-term care facilities, provide accommodation and care for those who can no longer live in their own home and need ongoing help with everyday tasks or complex health care [3]. Approximately 30% of all deaths in Australia occur in RAC homes, second only to hospitals. RAC homes are therefore increasingly involved in supporting older people at the end of life [4]. The provision of high-quality palliative care is important to support these residents who often have limited life expectancy and experience comorbid medical, cognitive and mental health conditions [5]. Consequently, palliative care is increasingly considered to be a core component of healthcare delivery in RAC homes [6].

Palliative care may be provided in RAC homes as generalist palliative care, delivered by aged care staff and general practitioners as part of routine care, or as specialist palliative care, involving professionals working solely in the field of palliative care, often in multi-disciplinary teams [7]. The implementation of palliative care in RAC has been shown to prevent unnecessary medical interventions, reduce physical burden, maintain bodily comfort and support dignified end-of-life transitions [8].

Despite its recognised clinical benefits, decisions about investing in palliative care must also take into account its economic implications. High-quality economic evidence regarding palliative care in RAC is therefore important for guiding well-informed decision-making about resource allocation [9]. Although existing systematic reviews have examined the cost-effectiveness of palliative care across multiple care settings, the economic evidence specific to RAC has not been synthesised separately [10, 11]. RAC homes operate within constrained budgets and face increasing financial pressures [12]. A clearer understanding of both the costs and cost-effectiveness of palliative care in RAC is necessary to support efficient resource allocation and maximise value for money [13, 14]. While cost analyses provide information on the resources required and the financial impact of palliative care service delivery, cost-effectiveness evaluations measure how efficiently these resources translate into improved resident outcomes and quality of care [15].

This review aims to map existing evidence on the costs and cost-effectiveness of palliative care delivered in RAC homes and to identify gaps in the current evidence base. The findings of this research are expected to inform decision-making regarding palliative care provision in RAC settings and offer insights for future research in this area.

## Methods

This review followed the Joanna Briggs Institute (JBI) method for scoping reviews [16] and was reported in accordance with the Preferred Reporting Items for Systematic Reviews and Meta-Analyses extension for Scoping Reviews guidelines [17] (see Supplementary Table S1 in the Supplementary Material). The protocol was originally registered in PROSPERO (CRD420251177461) as a systematic review; however, due to the broad research question, heterogeneous study designs and non-standardised outcomes, the review was subsequently conducted as a scoping review to map the available evidence. The search strategy, eligibility criteria and data extraction remain consistent with the original protocol. The research question was: what is the available evidence on the costs and cost-effectiveness of palliative care in residential aged care?

### Terminology

The terms ‘palliative care’, ‘hospice’ and ‘end-of-life care’ are used inconsistently in the literature and often overlap in both conceptualisation and practice. Given the aim of capturing a comprehensive body of evidence in this field, the term ‘palliative care’ has been adopted as an umbrella term in this review, encompassing palliative care, hospice care and end-of-life care. When reporting and interpreting individual studies, the original terminology used by the study authors has been retained.

### Search strategy

A preliminary search was conducted to identify relevant keywords, Medical Subject Headings and study designs. The draft search strategy was developed through discussion within the research team and subsequently reviewed by an experienced research librarian. The final search strategy included terms relating to [1] costs or cost-effectiveness, [2] palliative care [3] and residential aged care, using both controlled vocabulary and free-text terms. The full search terms are provided in Supplementary Table S2. We searched the five databases of PubMed, EMBASE, CINAHL, SCOPUS and Cochrane Central Register of Controlled Trials (CENTRAL). All searches were conducted in August 2025 (and updated in January 2026).

### Eligibility criteria

We included empirical studies of any design that reported quantitative economic outcomes related to palliative care delivered in residential aged care settings. There were no restrictions on language or year of publication. Studies comparing palliative care in residential aged care with care delivered in other settings were eligible if data specific to residential aged care could be extracted separately.

Studies were excluded if the intervention was not explicitly described by the authors as palliative care, end-of-life care or hospice care. Studies in which advance care planning was the primary intervention, rather than being delivered as part of a broader palliative care service or care model, were also excluded.

### Selection of studies

All titles and abstracts of articles returned from the formal search were uploaded to the online review management software, Covidence, which automatically removed duplicate records. After duplicate removal, two reviewers (X.Y. and F.L.) independently screened titles and abstracts against the predefined inclusion and exclusion criteria. Following initial screening, potentially relevant articles were subsequently assessed in full text for eligibility. At each stage of the screening, the two reviewers (X.Y. and F.L.) compared the results, with any disagreements resolved through discussion with a third reviewer (H.C.) for a final decision.

### Data extraction

A data extraction form was developed in Microsoft Excel in line with the research aims. Data were extracted from included studies by one reviewer (X.Y.) and independently checked by a second reviewer (F.L.). Extracted data were organised and summarised to describe study characteristics, costing methods and study findings across included studies. A full list of data fields that were extracted is provided in Supplementary Table S3 in the Supplementary Material.

### Reporting quality

The reporting quality of the included studies was assessed using the Consolidated Health Economic Evaluation Reporting Standards 2022 (CHEERS 2022) checklist to characterise the transparency and completeness of reporting [18].

### Data synthesis

Data synthesis was primarily descriptive and comparative. Consistent with the aims of this review, particular emphasis was placed on costing methods and study findings. Costing methods were analysed in terms of cost perspective, time horizon, cost components, data sources and analytical approach. Palliative care models evaluated in each study were classified into generalist palliative care, specialist palliative care, end-of-life care and hospice, according to the terminology and definitions used by the original authors. Study findings were grouped according to the type of economic outcome reported (care delivery costs, healthcare system costs and cost-effectiveness outcomes) and summarised descriptively.

## Results

### Eligible studies

**Figure 1:**
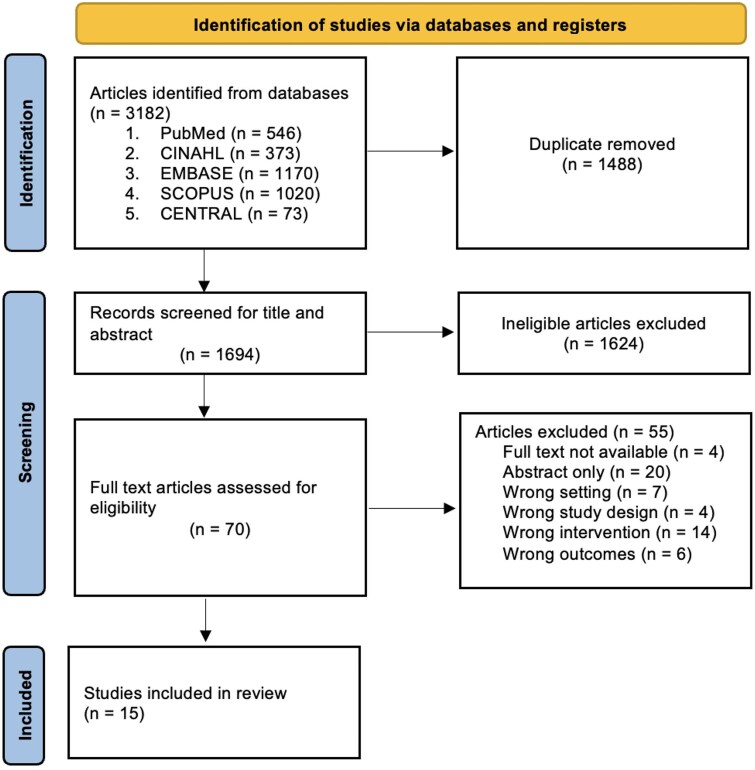
Study screening PRISMA flowchart.

The initial database search yielded 3182 records. After removing 1488 duplicates, 1694 unique records were screened, of which 1624 were excluded at the title and abstract screening stage. The remaining 70 records were retrieved for full-text assessment, of which 15 studies were included in the analysis. The full study selection process is presented in Figure 1.

### Study characteristics

The main characteristics of the 15 included studies are summarised in Table 1. Studies were published between 2004 and 2023, with the majority (*n* = 14) published after 2013. Geographically, five studies were conducted in North America [19–23], six in Europe [24–30], three in Oceania [30–32] and one in Asia [33].

**Table 1:** Study characteristics.

Author/year	Country	Study design	Type of economic evaluation	Population (*n* = sample size)	Intervention	Comparator
Miller et al. (2004)	United States	Retrospective cohort study	Cost analysis	NH residents (*n* = 5774).	Hospice	Usual care (non-hospice)
Simoens et al. (2013)	Belgium	Retrospective cohort study	Cost analysis	Terminal patients with chronic diseases (*n* = 181)	Palliative care	Usual care
Amador et al. (2014)	UK	Quasi-experimental pre-post study	Cost analysis	Older people with dementia (OPWD) residents in care homes (phase 1: *n* = 133, phase 2: *n* = 74)	A modified appreciative inquiry (AI) intervention: foster collaborative working among CH staff and visiting healthcare professionals to facilitate codevelopment of context-specific innovations to improve EOL care for older people with dementia	Usual care (pre-intervention)
Teo et al. (2014)	Singapore	Quasi-experimental study	Cost analysis	Residents identified to be at risk of dying within 1 year (*n* = 245)	Project CARE programme (Care at the End-of-Life for Residents in homes for the Elderly): provide end-of-life discussion through ACP and to improve palliative care in the voluntary welfare nursing homes	Usual care (without any end-of-life care programme)
Gozalo et al. (2015)	United States	Natural experiment	Cost analysis	All 2004 (baseline period) and 2009 nursing home decedents who were 67 years of age or older at death and who had fee-for-service Medicare for the last 2 years of life (*n* = 786,328)	Hospice expansion	Usual care (pre-hospice expansion)
Unroe et al. (2015)	United States	Retrospective cohort study	Cost analysis	Hospice patients aged 65 years and above from Wishard Health Services (*n* = 3771).	Hospice among nursing home	Hospice among non-nursing home
Unroe et al. (2016)	United States	Retrospective cohort study	Cost analysis	Long-stay (>90 days) NH decedents (*n* = 2510).	Hospice	Usual care (non-hospice)
Moore et al. (2017)	UK	Naturalistic feasibility study	Non-economic evaluation	Residents with advanced dementia (*n* = 30)	Compassion Intervention: (1) facilitation of an integrated, multidisciplinary approach to assessment, treatment and care; (2) Education, training and support for formal and informal carers	N/A
Chapman et al. (2018)	Australia	Quasi-experimental study	Non-economic evaluation	Residents of four residential facilities in (Canberra) Australia (*n* = 277)	An integrated model of proactive specialised palliative care: involved a palliative care nurse practitioner leading ‘Palliative Care Needs Rounds’ to support clinical decision-making, education and training	Usual care (general practitioners referred residents to specialist palliative care (SPC) on a case-by-case basis, without access to the clinical and educational intervention of palliative care needs rounds)
Bray et al. (2020)	UK	Cost modelling study	Cost analysis	Residents with advanced dementia in UK care homes (*n* not stated)	Namaste Care Intervention UK (NCI-UK): a standardised, evidence-based refinement of the original Namaste Care intervention, which is a multi-component intervention developed in the USA as a way of caring for people with advanced dementia	Usual care (not receiving Namaste Care)
El Alili et al. (2020)	Netherland	Stepped wedge cluster randomised controlled trial	Cost-effectiveness analysis	Residents with advanced dementia and their family caregivers (*n* = 231)	Namaste Care Family programme: a multidimensional care programme with psychosocial, sensory and spiritual components that incorporates tailored and personalised care until death for people with advanced dementia, involving their family caregivers in care activities and training sessions	Usual care (not restricted in any way)
Forbat et al. (2020)	Australia	Stepped-wedge cluster randomised controlled trial	Non-economic evaluation	Residents in 12 Australian care homes for older people (*n* = 1700)	Specialist palliative care needs rounds: a model of care providing specialist palliative care in care homes	Usual care (the specialist palliative care clinicians providing ad hoc reactive clinical consultations when referred by facility staff)
Wichmann et al. (2020)	Seven EU countries: Belgium, Finland, Italy, Netherlands, Poland, England and Switzerland	Parallel cluster randomised controlled trial	Cost-effectiveness analysis	Residents in long-term care facilities (Baseline: *n* = 551; Post-intervention: *n* = 983)	‘PACE Steps to Success’ intervention: integrate general palliative care into day-to-day routines in LTCFs by means of a train-the-trainer approach	Usual care (use all supportive services without restriction)
Comans et al. (2021)	Australia	Retrospective observational study	Cost analysis	People requiring palliative care at the end of life (*n* not stated)	A modified unit within a residential aged care facility (RACF) for people requiring palliative care at the end of life (Lavender Suite)	(i) A specialised palliative care unit within a hospital and (ii) A standard high-care RACF unit
Aldridge et al. (2023)	United States	Retrospective cohort study	Cost analysis	Medicare Current Beneficiary Survey (MCBS) participants who had dementia and who died during the period 2002–19 (*n* = 4621)	Hospice	Usual care (non-hospice)

Observational designs were most common (*n* = 6), all of which were retrospective. Quasi-experimental designs were also applied (*n* = 4). Three studies drew on data from cluster randomised controlled trials, with randomisation applied at the institution level. The remaining two studies included a naturalistic feasibility study and a cost modelling study. Studies were further categorised as full or partial economic evaluations following standard health economics definitions [34]. Only two were full economic evaluations comparing both the costs and effects [28, 29]. The majority (*n* = 10) were partial economic evaluations, primarily cost analyses comparing costs with and without palliative care. Three studies did not undertake formal economic evaluations: two evaluated changes in healthcare use and reported associated cost savings as secondary outcomes [30, 32] and one reported costs descriptively to inform feasibility and implementation [26].

All included studies focused on RAC residents, in line with the eligibility criteria. Within this population, five studies further specified residents with dementia [23, 25–28], and one specified residents with chronic disease [24], while the remaining nine studies did not specify a particular clinical subgroup. Regarding the interventions evaluated, end-of-life care was the most common (*n* = 6) [25–28, 31, 33], followed by hospice care (*n* = 5) [19–23]. Generalist palliative care [24, 29] and specialist palliative care [30, 32] were each evaluated in two studies. As for comparators, most studies compared palliative care with usual care (*n* = 12), whereas two studies compared costs across different settings [21, 31], and one study reported costs descriptively without a comparator [26].

### Costing methods

Of the 15 included studies, only five studies explicitly specified the costing perspective: two adopted a healthcare system perspective [29, 33], one adopted a healthcare payer perspective [24], one a societal perspective [28] and one a social care system perspective [31]. Seven studies did not report the costing perspective, and in the remaining three studies, perspective was not applicable, as they did not undertake formal economic evaluations [26, 30, 32]. Nine studies applied death-anchored time horizons, eight of which were within the last year of life [19, 20, 22–24, 29, 32, 33] and one measured costs from hospice election to death [21]. Five studies applied fixed time horizons, ranging from 3 to 12 months [25–28, 30].

**Figure 2:**
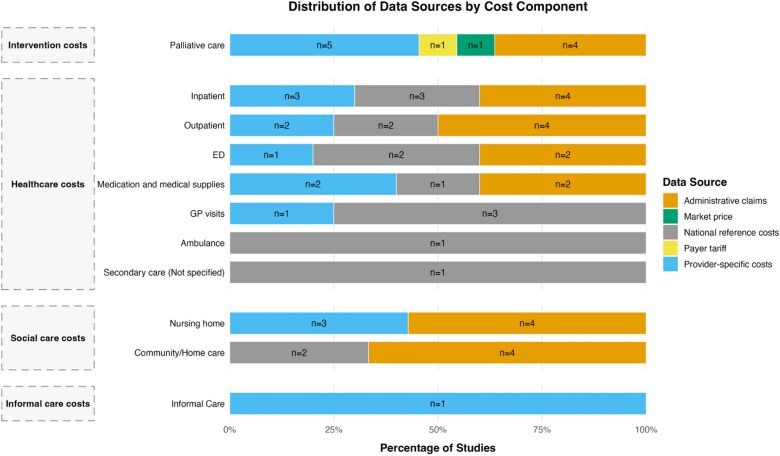
Data sources for costs across cost component categories.

Cost components varied across included studies and were categorised into intervention costs, healthcare costs, social care costs and informal care costs. Intervention costs refer to the costs of delivering palliative care and were explicitly included in 11 studies. Healthcare costs were reported with the widest range of cost items, with inpatient care (*n* = 10) and outpatient care (*n* = 8) the most commonly included components. Social care costs comprised nursing home costs (*n* = 7) and community/home care costs (*n* = 6). Informal care costs were rarely considered, with only one study including costs incurred by family caregivers [28]. Across studies, cost data were sourced from provider-specific costs, national reference costs, administrative claims, payer tariffs and market prices, with administrative claims used most commonly (Figure 2). Detailed costing methods for each study are provided in Supplementary Table S4 in the Supplementary Material.

### Study findings

**Figure 3:**
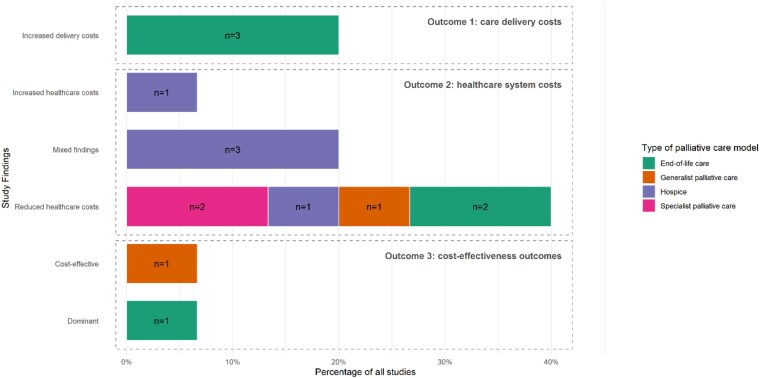
Study findings across included studies.

Overall, the included studies reported heterogeneous economic findings across different types of economic analyses. Findings were grouped into three broad categories: [1] care delivery costs, [2] healthcare system costs and [3] cost-effectiveness outcomes (Figure 3). A detailed breakdown of the cost outcomes reported in each study is provided in Supplementary Table S5 in the Supplementary Material.

Three studies focused exclusively on care delivery costs and reported additional costs associated with palliative care delivery in RAC settings [26, 27, 31].

Ten studies assessed healthcare utilisation and associated costs of palliative care. Of these, six studies reported reductions in healthcare costs, one reported increased healthcare costs and three reported mixed findings. Among the six studies reporting cost savings, two derived these savings from reductions in hospital admissions and length of stay without fully accounting for intervention costs [30, 32]. The remaining four studies employed comparative designs evaluating healthcare costs in both intervention and usual care groups [22, 24, 25, 33]. Across studies, reported cost-savings were primarily attributed to reductions in hospital resource use, including fewer hospital admissions, shorter lengths of stay, reduced transfers out of RAC and avoidance of acute care.

In contrast, one study reported an overall increase in healthcare expenditures associated with hospice expansion. The increase in total costs was driven by additional hospice service costs, which exceeded savings from reduced aggressive care near death and significant reductions in hospital transfer rates [20].

Three studies reported mixed cost findings. Two studies found cost savings only in the final weeks of life but higher or no difference in costs over longer time horizons [19, 23], while one study found higher costs among residents who experienced transitions between care settings compared with those receiving hospice exclusively in a single setting [21].

Only two studies conducted full economic evaluations, both reporting favourable conclusions. Wichmann *et al*. (2020) found a palliative care intervention to be cost-effective, with a statistically significant improvement in quality of dying and significant cost savings from decreased hospitalisation-related costs [29]. El Alili *et al*. (2020) reported the intervention to be dominant over usual care, although differences in costs and outcomes were not statistically significant [28].

### Reporting quality

**Figure 4:**
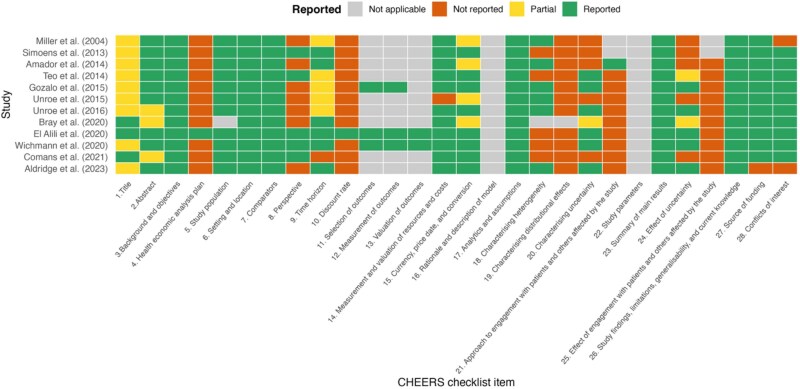
Reporting quality of included studies based on the CHEERS 2022 checklist. Note: Each row represents an individual study, and each column corresponds to a CHEERS item, with colours indicating whether an item was reported, partially reported, not reported or not applicable.

A summary of the CHEERS 2022 quality assessment is presented in Figure 4. Three studies that did not constitute economic evaluations were excluded from the assessment.

Overall, reporting quality varied across CHEERS items. Only two studies were full economic evaluations; therefore, CHEERS items 11–13 were not applicable to most included studies. Additionally, as none of the studies employed a decision-analytic modelling approach, items 16 and 22 were also not applicable [35]. Items related to the study context (e.g. background and objectives, setting and location and study population) were generally well reported. In contrast, items related to economic methods and analytical robustness, including perspective (item 8), time horizon (item 9), characterising uncertainty (item 20) and distributional effects (item 19), were frequently poorly reported or absent. Discounting was not applied across studies, which is consistent with the predominantly short time horizons (≤12 months), though only one study explicitly justified this decision [28].

## Discussion

This scoping review mapped the economic evidence on palliative care in residential aged care settings. The included studies showed substantial heterogeneity in study design, perspective, cost components and analytical methods. Despite this, most studies found reductions in hospital use associated with palliative care, including fewer hospital admissions and shorter lengths of stay, which led to lower total healthcare costs. This is consistent with findings from other settings [36, 37]. However, studies examining care delivery costs reported that delivering palliative care in RAC incurs additional costs. Whether these additional costs were offset by savings from reduced hospital use varied across studies.

Variations in findings may have been influenced by the time horizon of cost assessment. Across studies, cost-saving effects were more commonly observed when costs were assessed over shorter periods before death, such as the final month of life, whereas these effects were attenuated or no longer observed when longer time horizons, such as 6 months or 1 year before death, were applied. This is consistent with evidence from a recent meta-analysis, which found cost-saving effects of palliative care within the last six months of life, though savings were less evident over longer time horizons [38]. This may reflect the concentration of high-intensity hospital utilisation during the end-of-life period [39], when opportunities for reducing hospital use are greatest, while delivery costs accumulate over longer periods, progressively offsetting any savings achieved.

The findings should be interpreted in light of several methodological limitations of the included studies. The majority of studies were observational or quasi-experimental in design, with only three studies using randomised controlled trials. The non-randomised designs that characterised most included studies are particularly susceptible to selection bias. This is especially relevant for the studies comparing hospice users with non-users in the United States [19, 22, 23], where hospice enrolment is typically a voluntary election. Differences in disease trajectory, functional status and care preferences may therefore partly explain observed cost differences. In addition, several studies that reported favourable cost outcomes may overstate the economic impact. In two studies, cost savings attributed to reduced hospital use were derived without fully incorporating implementation costs, and therefore, may not reflect true net economic benefit [30, 32]. One economic evaluation concluded cost-effectiveness despite non-significant differences in both costs and outcomes [28], which may limit the strength of the economic conclusions drawn. In terms of costing methods, most studies relied on administrative claims data or national reference costs as valuation sources. Although commonly adopted in cost analyses, these valuation methods reflect reimbursement prices or average costs rather than true opportunity costs, and therefore, may not fully capture underlying resource consumption [40]. Furthermore, substantial variation in currencies, price years, cost perspectives, cost components and intervention types across studies limited the comparability of absolute cost estimates and precluded meaningful cross-study comparisons.

### Implications for future research

Three important gaps in the existing evidence base were identified, each with implications for future research. First, most included studies focused primarily on cost outcomes, without incorporating patient-centred outcomes that are essential for assessing the full economic value of palliative care. While palliative care has been shown to improve patient outcomes, in particular symptom control, quality of life and patient and family satisfaction [41], these benefits were not captured in studies reporting costs alone. Only two studies conducted full economic evaluations considering both costs and patient-centred outcomes, and the scarcity of such evidence constrains conclusions regarding whether palliative care represents good value for money in RAC settings. There is a clear need for rigorous economic evaluations that incorporate patient-centred outcomes alongside costs. Given the inconsistencies in reporting identified across the included studies, adherence to CHEERS guidelines would support improved transparency and completeness in future economic evaluations.

Second, all included studies assessed costs over relatively short time horizons, most within one year and typically anchored to death. Given the evolution of palliative care from an end-of-life only approach to a needs-based model that provides targeted care and holistic symptom relief early in the course of the disease [42, 43], economic evidence extending beyond the end-of-life period is needed. Earlier integration of palliative care may improve symptom management and quality of life over a longer period [44], yet its implications for long-term economic outcomes in residential aged care remain unclear. Future research should therefore evaluate the long-term costs and outcomes associated with earlier integration of palliative care across the disease trajectory.

Third, residents in RAC homes often present with complex care needs, including advanced dementia and multiple comorbidities [45], which may require the involvement of specialist palliative care services [46, 47]. There has also been increasing clinical and policy interest in integrating specialist palliative care into residential aged care settings to manage the complex medical needs [48]. However, no studies included in this review conducted formal economic evaluations of specialist palliative care in RAC settings. The two studies that examined specialist palliative care reported cost-related outcomes as secondary findings only, without incorporating a comprehensive cost assessment or patient-centred outcomes [30, 32]. Future economic evaluations of specialist palliative care in RAC settings are therefore needed to inform service planning and resource allocation.

### Strengths and limitations

To our knowledge, this is the first review mapping the existing evidence on the costs and cost-effectiveness of palliative care delivered in RAC settings. The review encompassed a broad range of economic evidence, thereby capturing multiple dimensions of the economic impact of palliative care in this context.

Nevertheless, several limitations should be acknowledged. Firstly, consistent with scoping review methodology, findings were synthesised using a descriptive approach that does not incorporate study quality or allow for estimation of overall effect sizes. This limits the ability to draw robust conclusions regarding the magnitude and consistency of cost outcomes across studies. Secondly, inclusion in this review was restricted to interventions explicitly described by the original study authors as palliative care. Although this ensured consistency with how palliative care was defined and reported in the existing literature, it may have resulted in the exclusion of interventions that incorporated palliative principles but were described using alternative labels or frameworks, potentially narrowing the scope of the evidence captured. Additionally, this review examined palliative care as an integrated model of care rather than focusing on individual components or symptom-specific interventions. Accordingly, studies evaluating isolated elements of palliative care, such as advanced care planning only, were not included. This approach was intentionally adopted to reflect palliative care as a holistic model, consistent with how palliative care is typically delivered in residential aged care settings. By providing a common analytical basis for assessing palliative care as a comprehensive model of care, this review aimed to capture its overall economic implications. However, this decision also limits the ability to investigate the relative cost and effectiveness contributions of specific components within palliative care models.

## Conclusion

Existing evidence suggests that palliative care in RAC may be associated with reductions in healthcare costs, primarily driven by reduced hospital admissions and shorter length of stay. However, most included studies were partial economic evaluations with heterogeneous methods, perspectives and cost components, limiting the strength of conclusions that can be drawn.

Overall, the economic evidence remains limited, particularly with respect to robust cost-effectiveness analyses. Future research should prioritise high-quality economic evaluations that comprehensively capture healthcare and social care costs while incorporating patient-centred outcomes to better assess the value of palliative care and inform resource allocation in residential aged care.

## Supplementary Material

aa-26-0719-File002_afag217
