# Supplementary material for: Costs and cost-effectiveness of palliative care in residential aged care homes: a scoping review
Source: Age Ageing. 2026 Jul 24;55(7):afag217. doi: 10.1093/ageing/afag217 (PMC13398384; doi:10.1093/ageing/afag217)
Supplement: aa-26-0719-File002_afag217 [file aa-26-0719-file002_afag217.docx]

**Costs and Cost-Effectiveness of Palliative Care in Residential Aged Care Homes: A Scoping Review**

**Supplementary material**

Contents

[Table S1. PRISMA-ScR Checklist 2](#_Toc232157098)

[Table S2. Search Strategy 4](#_Toc232157099)

[Table S3. Full list of data fields extracted 5](#_Toc232157100)

[Table S4. Costing methods by study 6](#_Toc232157101)

[Table S5. Cost outcomes reported by study 10](#_Toc232157102)

## Table S1. PRISMA-ScR Checklist

| **SECTION** | **ITEM** | **PRISMA-ScR CHECKLIST ITEM** | **REPORTED ON PAGE #** |
| --- | --- | --- | --- |
| **TITLE** | | | |
| Title | 1 | Identify the report as a scoping review. | 1 |
| **ABSTRACT** | | | |
| Structured summary | 2 | Provide a structured summary that includes (as applicable): background, objectives, eligibility criteria, sources of evidence, charting methods, results, and conclusions that relate to the review questions and objectives. | 1 |
| **INTRODUCTION** | | | |
| Rationale | 3 | Describe the rationale for the review in the context of what is already known. Explain why the review questions/objectives lend themselves to a scoping review approach. | 2-3 |
| Objectives | 4 | Provide an explicit statement of the questions and objectives being addressed with reference to their key elements (e.g., population or participants, concepts, and context) or other relevant key elements used to conceptualize the review questions and/or objectives. | 2 |
| **METHODS** | | | |
| Protocol and registration | 5 | Indicate whether a review protocol exists; state if and where it can be accessed (e.g., a Web address); and if available, provide registration information, including the registration number. | 3 |
| Eligibility criteria | 6 | Specify characteristics of the sources of evidence used as eligibility criteria (e.g., years considered, language, and publication status), and provide a rationale. | 3 |
| Information sources* | 7 | Describe all information sources in the search (e.g., databases with dates of coverage and contact with authors to identify additional sources), as well as the date the most recent search was executed. | 3 |
| Search | 8 | Present the full electronic search strategy for at least 1 database, including any limits used, such that it could be repeated. | Supplementary Table S2 |
| Selection of sources of evidence† | 9 | State the process for selecting sources of evidence (i.e., screening and eligibility) included in the scoping review. | 4 |
| Data charting process‡ | 10 | Describe the methods of charting data from the included sources of evidence (e.g., calibrated forms or forms that have been tested by the team before their use, and whether data charting was done independently or in duplicate) and any processes for obtaining and confirming data from investigators. | 3-4 |
| Data items | 11 | List and define all variables for which data were sought and any assumptions and simplifications made. | Supplementary Table S3 |
| Critical appraisal of individual sources of evidence§ | 12 | If done, provide a rationale for conducting a critical appraisal of included sources of evidence; describe the methods used and how this information was used in any data synthesis (if appropriate). | 4 |
| Synthesis of results | 13 | Describe the methods of handling and summarizing the data that were charted. | 4 |
| **RESULTS** | | | |
| Selection of sources of evidence | 14 | Give numbers of sources of evidence screened, assessed for eligibility, and included in the review, with reasons for exclusions at each stage, ideally using a flow diagram. | 4-5 |
| Characteristics of sources of evidence | 15 | For each source of evidence, present characteristics for which data were charted and provide the citations. | 5 |
| Critical appraisal within sources of evidence | 16 | If done, present data on critical appraisal of included sources of evidence (see item 12). | 12 |
| Results of individual sources of evidence | 17 | For each included source of evidence, present the relevant data that were charted that relate to the review questions and objectives. | 11 |
| Synthesis of results | 18 | Summarize and/or present the charting results as they relate to the review questions and objectives. | 10, Figure 2  13, Figure 3 |
| **DISCUSSION** | | | |
| Summary of evidence | 19 | Summarize the main results (including an overview of concepts, themes, and types of evidence available), link to the review questions and objectives, and consider the relevance to key groups. | 13 |
| Limitations | 20 | Discuss the limitations of the scoping review process. | 15 |
| Conclusions | 21 | Provide a general interpretation of the results with respect to the review questions and objectives, as well as potential implications and/or next steps. | 15 |
| **FUNDING** | | | |
| Funding | 22 | Describe sources of funding for the included sources of evidence, as well as sources of funding for the scoping review. Describe the role of the funders of the scoping review. | 16 |

JBI = Joanna Briggs Institute; PRISMA-ScR = Preferred Reporting Items for Systematic reviews and Meta-Analyses extension for Scoping Reviews.

* Where *sources of evidence* (see second footnote) are compiled from, such as bibliographic databases, social media platforms, and Web sites.

† A more inclusive/heterogeneous term used to account for the different types of evidence or data sources (e.g., quantitative and/or qualitative research, expert opinion, and policy documents) that may be eligible in a scoping review as opposed to only studies. This is not to be confused with *information sources* (see first footnote).

‡ The frameworks by Arksey and O’Malley (6) and Levac and colleagues (7) and the JBI guidance (4, 5) refer to the process of data extraction in a scoping review as data charting*.*

§ The process of systematically examining research evidence to assess its validity, results, and relevance before using it to inform a decision. This term is used for items 12 and 19 instead of "risk of bias" (which is more applicable to systematic reviews of interventions) to include and acknowledge the various sources of evidence that may be used in a scoping review (e.g., quantitative and/or qualitative research, expert opinion, and policy document).

*From:* Tricco AC, Lillie E, Zarin W, O'Brien KK, Colquhoun H, Levac D, et al. PRISMA Extension for Scoping Reviews (PRISMAScR): Checklist and Explanation. Ann Intern Med. 2018;169:467–473. [doi: 10.7326/M18-0850](http://annals.org/aim/fullarticle/2700389/prisma-extension-scoping-reviews-prisma-scr-checklist-explanation).

## Table S2. Search Strategy

Searches were last performed on the 7th of January 2026.

| **No.** | **PubMed** |
| --- | --- |
| #1 | "Homes for the Aged"[MeSH] OR "Nursing Homes"[MeSH] OR "residential aged care"[tiab] OR "aged care home*"[tiab] OR "aged care facilit*"[tiab] OR "care home*"[tiab] OR "nursing home*"[tiab] OR "long term care home*"[tiab] OR "long-term care home*"[tiab] OR "long term care facilit*"[tiab] OR "long-term care facilit*"[tiab] OR "residential care"[tiab] OR "residential home"[tiab] OR "residential facilit*"[tiab] OR "skilled nursing facilit*"[tiab] |
| #2 | "Palliative Care"[MeSH] OR "Hospice Care"[MeSH] OR "Terminal Care"[MeSH] OR "Palliative Medicine"[MeSH] OR Palliati*[tiab] OR "Comfort Care"[tiab] OR hospice[tiab] OR "Terminal Care"[tiab] OR "last year of life"[tiab] OR "end of life"[tiab] OR "end-of-life"[tiab] |
| #3 | "Costs and Cost Analysis"[Mesh] OR "Health Care Costs"[MeSH] OR cost*[tiab] OR economic*[tiab] OR expenditure*[tiab] |
| #4 | #1 AND #2 AND #3 |

| **No.** | **EMBASE** |
| --- | --- |
| #1 | 'home for the aged'/exp OR 'nursing home'/exp OR 'residential home'/exp OR 'residential aged care':ti,ab OR 'aged care home*':ti,ab OR 'aged care facilit*':ti,ab OR 'care home*':ti,ab OR 'nursing home*':ti,ab OR 'long term care home*':ti,ab OR 'long-term care home*':ti,ab OR 'long term care facilit*':ti,ab OR 'long-term care facilit*':ti,ab OR 'residential care':ti,ab OR 'residential home':ti,ab OR 'residential facilit*':ti,ab OR 'skilled nursing facilit*':ti,ab |
| #2 | 'palliative therapy'/exp OR 'terminal care'/exp OR 'palliative nursing'/exp OR palliati*:ti,ab OR 'comfort care':ti,ab OR hospice:ti,ab OR 'terminal care':ti,ab  OR 'last year of life':ti,ab OR 'end of life':ti,ab OR 'end-of-life':ti,ab |
| #3 | 'economic evaluation'/exp OR 'health care cost'/exp OR cost*:ti,ab OR expenditure*:ti,ab OR economic*:ti,ab |
| #4 | #1 AND #2 AND #3 |

| **No.** | **CINAHL（via EBSCOhost）** |
| --- | --- |
| #1 | (MH "Residential Care+") OR (MH "Residential Facilities+")  OR (TI "residential aged care" OR AB "residential aged care") OR (TI "aged care home*" OR AB "aged care home*") OR (TI "aged care facilit*" OR AB "aged care facilit*") OR (TI "care home*" OR AB "care home*") OR (TI "nursing home*" OR AB "nursing home*") OR (TI "long term care home*" OR AB "long term care home*") OR (TI "long-term care home*" OR AB "long-term care home*") OR (TI "long term care facilit*" OR AB "long term care facilit*") OR (TI "long-term care facilit*" OR AB "long-term care facilit*") OR (TI "residential care" OR AB "residential care") OR (TI "residential home*" OR AB "residential home*") OR (TI "residential facilit*" OR AB "residential facilit*") OR (TI "skilled nursing facilit*" OR AB "skilled nursing facilit*") |
| #2 | (MH "Palliative Care+") OR (MH "Terminal Care+") OR (MH "Palliative Medicine+")  OR (TI "palliati*" OR AB "palliati*") OR (TI "comfort care" OR AB "comfort care")  OR (TI "hospice" OR AB "hospice") OR (TI "terminal care" OR AB "terminal care")  OR (TI "end of life" OR AB "end of life") OR (TI "end-of-life" OR AB "end-of-life") OR (TI "last year of life" OR AB "last year of life") |
| #3 | (MH "Costs and Cost Analysis+") OR (TI cost* OR AB cost*) OR (TI expenditure* OR AB expenditure*) OR (TI economic* OR AB economic*) |
| #4 | #1 AND #2 AND #3 |

| **No.** | **CENTRAL** |
| --- | --- |
| #1 | [mh "Homes for the Aged"] OR [mh "Nursing Homes"] OR "residential aged care":ti,ab OR (aged NEXT care NEXT home*):ti,ab OR (aged NEXT care NEXT facilit*):ti,ab OR (care NEXT home*):ti,ab OR (nursing NEXT home*):ti,ab OR (long NEXT term NEXT care NEXT home*):ti,ab OR (long NEXT term NEXT care NEXT facilit*):ti,ab OR "residential care":ti,ab OR "residential home":ti,ab OR (residential NEXT facilit*):ti,ab OR (skilled NEXT nursing NEXT facilit*):ti,ab |
| #2 | [mh "Palliative Care"] OR [mh "Hospice Care"] OR [mh "Terminal Care"] OR palliati*:ti,ab OR "comfort care":ti,ab OR hospice:ti,ab OR "terminal care":ti,ab OR "last year of life":ti,ab OR "end of life":ti,ab OR "end-of-life":ti,ab |
| #3 | [mh "Costs and Cost Analysis"] OR [mh "Health Care Costs"] OR cost*:ti,ab OR economic*:ti,ab OR expenditure*:ti,ab |
| #4 | #1 AND #2 AND #3 |

| **No.** | **SCOPUS** |
| --- | --- |
| #1 | TITLE-ABS-KEY("residential aged care") OR TITLE-ABS-KEY("aged care home*")  OR TITLE-ABS-KEY("aged care facilit*") OR TITLE-ABS-KEY("care home*")  OR TITLE-ABS-KEY("nursing home*") OR TITLE-ABS-KEY("long term care home*") OR TITLE-ABS-KEY("long-term care home*") OR TITLE-ABS-KEY("long term care facilit*") OR TITLE-ABS-KEY("long-term care facilit*") OR TITLE-ABS-KEY("residential care") OR TITLE-ABS-KEY("residential home") OR TITLE-ABS-KEY("residential facilit*") OR TITLE-ABS-KEY("skilled nursing facilit*") |
| #2 | TITLE-ABS-KEY("palliative care") OR TITLE-ABS-KEY("terminal care") OR TITLE-ABS-KEY(palliati*) OR TITLE-ABS-KEY("comfort care") OR TITLE-ABS-KEY(hospice) OR TITLE-ABS-KEY("last year of life") OR TITLE-ABS-KEY("end of life") OR TITLE-ABS-KEY("end-of-life") |
| #3 | TITLE-ABS-KEY(cost*) OR TITLE-ABS-KEY(economic*) OR TITLE-ABS-KEY(expenditure*) |
| #4 | #1 AND #2 AND #3 |

## Table S3. Full list of data fields extracted

| Data category | Full list |
| --- | --- |
| Study characteristics | Author/Year, Country, Study Design, Type of Economic Evaluation, Population, Sample Size, Intervention, Comparator |
| Costing methods | Year & Currency, Perspective, Time Horizon, Costs measured, Costs included, Costing Method, Data Source |
| Cost results | Cost components, Costing Method, Data Source, Total costs(values), Cost difference between intervention and control group, Cost impact |
| Study findings | The presented cost outcome measures, Cost impact, Main results, Key findings, Policy implications |

## Table S4. Costing methods by study

| **Study/Author** | **Cost components** | **Costing Method** | **Data Source** |
| --- | --- | --- | --- |
| [Miller et al. (2004)](https://agsjournals.onlinelibrary.wiley.com/doi/abs/10.1111/j.1532-5415.2004.52357.x) | Acute Inpatient, Nursing Home, Hospice, and other expenditure | claims-based costing | Medicare inpatient, SNF, hospice, hospital outpatient, and home health care standard analytic ﬁles were used. |
| [Simoens et al. (2013)](https://link.springer.com/article/10.1007/s10198-012-0384-9) | **Fixed nursing home costs:** Nursing staff, Energy, Pharmacy, Technical devices, Nourishment | Annual fixed nursing home costs were divided by the number of lay days. Fixed costs per patient were added to medical fees, pharmacy charges, and other charges to obtain a total cost per patient during a patient’s stay in a nursing home. | Accounting system |
|  | **Invoices by nursing homes to patients:** Medical fees (outpatient physician consultations), pharmacy, other charges (i.e., additional nourishment, ambulatory care) |  | Invoices provided data on charges |
|  | **Fixed hospital costs:** Hospital staff, Energy, Infrastructure, Nourishment, Medical fees (physician consults, labs, clinical biology), Pharmacy, Telephone, Single-room additional cost | The mean hospital costs per patient per day were multiplied by the number of days that a patient stayed in hospital. | Belgian hospital FINHOSTA accounting system |
|  | **Charges incurred by the NIHDI or by the patient (as reflected in hospital invoices)** medical fees, pharmacy, other charges (i.e., telephone costs and additional costs for a single room). |  | Invoice data reflected charges |
| [Amador et al. (2014)](https://onlinelibrary.wiley.com/doi/10.1002/gps.4061) | Hospital costs including ambulance service use costs, A and E visit costs, inpatient stay costs, outpatient visit costs); Community health care costs including district nurse visit costs, out-of-hours GP visit costs | unit costs * × service use | Unit cost data were taken from the Personal Social Services Research Unit volume for 2010, and medication costs from the British National Formulary. |
| [Teo et al. (2014)](https://journals.sagepub.com/doi/10.1177/0269216314526270?url_ver=Z39.88-2003&rfr_id=ori:rid:crossref.org&rfr_dat=cr_pub%20%200pubmed) | Hospitalisation, ED, SOC and polyclinic costs | Hospitalisation costs were estimated by multiplying the average cost per patient day by the inpatient LOS. Direct medical costs for ED, SOC and primary care visits were derived by multiplying the standardised unit cost by the number of visits to each level of care. | The average cost per patient day of hospitalisation was provided by the NHG finance department. Unit cost of ED, SOC, polyclinic estimates included manpower, medication and allocated fixed cost were obtained from the NHG finance department. |
|  | Nursing home cost | The unit cost in the nursing home was estimated by dividing the total expenses by the total number of nursing home residents. Cost per day stay at the nursing home was calculated by dividing the unit cost by 365 days. The total cost for a resident was calculated by the LOS of the nursing home multiplied by the cost per day stay. | Annual reports of each nursing home |
|  | Project CARE cost: Fixed cost consisted of manpower, transport, overheads and depreciation costs. Variable costs included palliative care visits by physicians, nurses and transport required for nursing home visits. | Fixed costs were attributed to individual patients by dividing the total fixed cost by the total number of nursing home residents. Per man-hour utilisation of each personnel was multiplied by the hourly wage rate to derive the total cost per visit. The sum of fixed cost per resident and variable cost per resident was the total cost per resident of Project CARE utilisation. | The expenditure of the Project CARE team was recorded on a quarterly basis. |
| [Gozalo et al. (2015)](https://www.nejm.org/doi/10.1056/NEJMsa1408705) | Inpatient, outpatient, post acute, home health, physician-visit, and hospice, | / | Medicare claims |
| [Unroe et al. (2015)](https://link.springer.com/article/10.1007/s11606-014-3080-x) | Not reported | Not reported | Medicare claims, Indiana Medicaid claims, and Minimum Data Set (MDS) reports |
| [Unroe et al. (2016)](https://agsjournals.onlinelibrary.wiley.com/doi/full/10.1111/jgs.14070?) | Total Medicare costs include hospice costs, inpatient costs, SNF costs and costs. Other includes durable medical equipment, emergency department, home health, outpatient | Costs are defined as the payment amounts obtained from Medicare and Medicaid claims data. The medical care component of the Consumer Price Index was used to account for inflation over the observation period. | Electronic medical records from Wishard Health Services merged with Medicare claims, Indiana Medicaid claims, and Minimum Data Set (MDS) version 2.0 reports. |
| [Moore et al. (2017)](https://pubmed.ncbi.nlm.nih.gov/28694253/) | The time the ICL spent on various activities was used to calculate the costs of Implementation | total ICL hours spent on different activities * tariff | The ICL kept a daily log of time spent on tasks related to implementation to enable estimation of costs. ICL hours spent on activities associated with the implementation were costed using the Department of Health and Health Education England tariffs. |
| [Chapman et al. (2018)](https://spcare.bmj.com/content/8/1/102) | Hospital bed day cost | Cost saving = reduction in length of stay* hospital bed day cost | The analysis of average bed day cost was calculated at $A918, based on data from hospital providers in 2014 (ACT Health, personal communication, 2015). |
| [Bray et al. (2020)](https://www.intpsychogeriatrics.org/article/S1041-6102(24)03686-X/fulltext) | Staff costs | An hourly rate of pay for each staff type and grade was calculated using the final salary figures, adjusted for holiday allocation and hours worked per week. | average salary information was obtained from a recruitment website (www.indeed.co.uk) |
|  | Capital costs | Prices for room hire were converted into hourly costs using an assumption of an 8-hour day. | Internet searches identified a range of prices for room hire in care homes or related care settings such as hospices or day centres |
|  | Consumable costs | a standard approach was used to create a complete set of consumable costs for all resources | A sample of eight care home staff was asked where they buy resources. This was supplemented by an internet search to identify care home suppliers who could provide national prices. The identified websites were searched systematically to find a minimum of six to eight prices for each item. |
| [El Alili et al. (2020)](https://pmc.ncbi.nlm.nih.gov/articles/PMC7473814/) | Healthcare costs include medication, hospitalisation, and emergency room and specialist visits. | Healthcare utilization was valued using standard costs from the Dutch costing guideline. Medication costs were valued using prices from the Royal Dutch Pharmacists Association. | questionnaires primarily based on a Dutch standardized data collection tool for older nursing home residents, the TOPIC-MDS |
|  | The Namaste Care Family program costs included costs of supplies for the intervention, any change (increase or decrease) in nursing staff time aswell as hiring extra nursing staff, and family and volunteer time investments. | Costs related to supplies and other investments, as well as actual costs of donated items, were collected by asking the participating nursing homes to estimate the monetary value of supplies and donations they received. Extra staff costs were estimated using their hourly wage. |  |
|  | Family costs included time spent with the participant, administrative tasks for the participant, travel time and distance to visit the participant, finding replacement for daily activities when visiting, and lost productivity due to family caregivers’ absenteeism from work. | The shadow price of these time investments is assumed to be equal to the tariff for cleaning work. Lost productivity costs due to absenteeism from work were calculated using gender-specific income values of the Dutch population. |  |
| [Forbat et al. (2020)](https://journals.sagepub.com/doi/10.1177/0269216319891077) | cost of hospitalisations | hospital bed-day cost × length of stay (LOS) | The hospital bed cost was calculated based on the most recent National Hospital Cost Data Collection Report 2015–2016. |
|  | Intervention costs | annual salaries | N/A |
| [Wichmann et al. (2020)](https://bmcmedicine.biomedcentral.com/articles/10.1186/s12916-020-01720-9#citeas) | hospital admissions: Night geriatric ward, Night psychiatric ward, Night internal medicine, Night surgery ward, Night neurology ward, Night general ward, Night intensive care unit, Emergency unit  Palliative care unit | Quantities of resources used in the last month of life (hospital admissions, visits of health care professionals, received intensive treatments) were multiplied by standard unit costs in euros. | **Quantities of resources use:** A cross-sectional study of resident deaths in participating LTCFs was conducted. For each case, multiple structured after-death questionnaires were filled in by a staff member most involved (preferably a nurse, otherwise a care assistant) and a relative.   These **unit costs** were based on reference prices from 2017, calculated by the Dutch National Health Care Institute (ZN) and average per unit sales prices established by the Dutch Health Care Authority (NZA). If prices were only available for other years, they were adjusted using the consumer price index published by Statistics Netherlands. If they were not available, health insurer contract rates were used. |
|  | visits of health care professionals: General practitioner, Geriatrician, Neurologist, Psychiatrist, Occupational therapist, Social worker, Psychologist, Physiotherapist |  |  |
|  | received intensive treatments like CPR or surgery: CPR, Artificial ventilation, Blood transfusion, Chemo or radiotherapy, Surgery |  |  |
|  | costs associated with the intervention: coordinators, trainers, materials, and accommodation | Mean costs associated with the intervention (hours dedicated to the intervention of coordinators and trainers, materials, accommodation) were identified and calculated back to resident level by multiplying the LTCFs’ number of beds by the occupancy rate. |  |
| [Comans et al. (2021)](https://onlinelibrary.wiley.com/doi/full/10.1111/ajag.12843) | Lavender Suite costs, including one site labour(RN and CW cost, Clinical care coordinator, Preadmission assessment, Training cost), medical visits, MDT team meeting cost, medication, and cost per bed day | The total number of resident days (per year) was calculated using the average remaining life expectancy of people who enter the Lavender Suite and the average occupancy rate. The cost per person day was calculated as annual (total) operating cost for the Lavender Suite divided by the total number of resident days. | Unit costs were based on actual data sourced from the facility, excluding the cost of a general practitioner appointment, for which the Australian Medicare schedule item level B was used. |
|  | Hospital-based Palliative Care Unit costs, including Transport (to get patients for tests, procedures and services), Ambulance, Staffing(Nursing, Medical  Allied Health), Medication, Radiology, Bed cost (overhead, electricity, cleaning, food etc.) | The Palliative Care Unit services on average 346 (inpatient) cases per year. Costs were broken down into transport, ambulance, nursing, medical, allied health, medication and radiology items. The total cost of the facility for the year was divided by the beds and days occupied to calculate an average per bed day cost. | Palliative Care Unit costs were obtained from a specialised, comprehensive 20-bed palliative care service within a 72-bed community hospital in Sydney, Australia. The hospital provides both inpatient, outpatient and community assessments for patients diagnosed with a life-threatening illness. |
|  | High Care in Residential Care Facility costs, including Staffing / Labour costs(Morning shift, Afternoon shift, Night shift),Bed cost (overhead, electricity, cleaning, food etc)" | The cost was calculated as annual (total) operating cost for a high-care unit divided by the total number of resident days (which equals the number of beds in the unit, average 30 residents multiplied by 365). | The data was sourced using both bottom-up and top-down measures and data collected from the same RACF that hosts the Lavender Suite. The unit costs for nurses and care workers were therefore the same for both high-care and Lavender Suite. |
| [Aldridge et al. (2023)](https://pmc.ncbi.nlm.nih.gov/articles/PMC12366792/) | Total health care costs were measured as as the sum of family out-of-pocket, Medicare, Medicare Advantage, Medicaid, private insurance, private health maintenance organization, Veterans Affairs, and other spending. | Expenditures were measured for each day of each person’s last 365 days of life. Expenditures were then aggregated for the last three days, one week, two weeks, one month, three months, and six months of life. All costs were adjusted for inflation to 2020 dollars. | Medicare Current Beneficiary Survey data from the period 2002–19 |

## Table S5. Cost outcomes reported by study

| **Study/Author** | **Year & Currency** | **Cost outcomes** | **Cost estimates** | | |
| --- | --- | --- | --- | --- | --- |
|  |  |  | **Subgroups** | **Intervention group** | **Control group** |
| [Miller et al. (2004)](https://agsjournals.onlinelibrary.wiley.com/doi/abs/10.1111/j.1532-5415.2004.52357.x) | 1999, USD | Average Expenditures in the Last Month of Life | Cancer/Short NH stay | 9,742 ± 5,327 | 12,465 ± 10,515 |
|  |  |  | Cancer / Long NH Stay | 7,090 ± 3,102 | 8,747 ± 9,184 |
|  |  |  | Dementia / Short NH Stay | 8,489 ± 4,790 | 11,025 ± 8,913 |
|  |  |  | Dementia / Long NH Stay | 6,768 ± 3,833 | 6,324 ± 6,327 |
|  |  |  | Other / Short NH Stay | 8,370 ± 4,609 | 8,370 ± 4,609 |
|  |  |  | Other / Long NH Stay | 6,917 ± 3,515 | 6,447 ± 6,952 |
|  |  | Government Expenditures in Last Month of life | Short-stay | 8,722±4,869 | 11,764±9,459 |
|  |  |  | Long-stay | 6,869±3,618 | 6,495±6,823 |
|  |  |  | Overall | 7,365±4,073 | 8,134±8,114 |
|  |  | Government Expenditures in Last 6 Months of life | Short-stay | 33,901±15,064 | 37,350±23,316 |
|  |  |  | Long-stay | 31,624±14,350 | 24,999±14,844 |
|  |  |  | Overall | 32,233±14,574 | 28,840±18,823 |
| [Simoens et al. (2013)](https://link.springer.com/article/10.1007/s10198-012-0384-9) | 2007/2008, EUR | Costs per patient during the final month of life | Overall | 2,456 ± 2,117 | 3,822 ± 3,232 |
| Amador et al. (2014) | 2010, GBP | Total costs per resident per month | Overall | 2577.72 | 2802.17 |
| Teo et al. (2014) | 2011, SGD | Mean costs in the last month | Overall | SGD$3998 | SGD$7701 |
|  |  | Mean costs in the last 3 months | Overall | SGD$8617 | SGD$15,746 |
| Gozalo et al. (2015) | 2007, USD | Medicare expenditure in the last year of life | Overall | G2004: $30,636; G2009: $40,542 | G2004: $36,745; G2009: $39,888 |
|  |  |  | Decedents with advanced dementia | G2004: $18,705; G2009: $30,481 | G2004: $22,521; G2009: $24,322 |
| Unroe et al. (2015) | Year not specified, USD | Combined costs to Medicare and Medicaid | Shorter hospice stays (less than a week) | Nursing-home-only: $251 | Crossover: $498 Near-transition: $488 Non-nursing home: $324 |
|  |  |  | 91–180 day hospice stays | Nursing-home-only: $285 | Crossover: $221,  Near-transition: $192 Non-nursing home: $147 |
| Unroe et al. (2016) | 2009, USD | Total Medicare Costs | 2 days before death | 791 ± 2,399 | 2,785 ± 6,018 |
|  |  |  | 7 days before death | 2,060 ± 6,120 | 5,231 ± 9,445 |
|  |  |  | 14 days before death | 3,436 ± 10,614 | 7,349 ± 12,108 |
|  |  |  | 30 days before death | 6,361 ± 13,804 | 10,677 ± 15,688 |
|  |  |  | 90 days before death | 16,156 ± 18,659 | 19,794 ± 27,750 |
|  |  |  | 180 days before death | 30,239 ± 23,234 | 32,501 ± 43,882 |
| Moore et al. (2017) | Year not specified, GBP | Costs of an interdisciplinary care leader (ICL) for 6 months | Overall | £18,255 | / |
| Chapman et al. (2018) | AUD, 2014 | Cost savingsfrom reduction in length of stay for 3 months | Overall | Not reported | Not reported |
| Bray et al. (2020) | Year not specified, GBP | Total cost per session | Overall | £237.78, £267.04, £331.65 | £168.38, £194.75 , £254.47 |
|  |  | Total session cost per resident | Overall | £29.72, £33.38, £41.46 | £21.05, £24.34, £31.81 |
| El Alili et al. (2020) | 2018, EUR | mean costs per 12 months | Overall | 7173 | 7484 |
| Forbat et al. (2020) | 2015–16, AUD | average monthly cost | Overall | $35,106 | $49,980 |
|  |  | Annual cost across 12 sites | Overall | $5,055,225 | $7,197,151 |
| Wichmann et al. (2020) | 2017, EUR | Costs in the last month of life | Overall | Baseline: €1,667.87 Post-intervention:€1,410.35 | Baseline: €1,361.89 Post-intervention: €1,962.64 |
| Comans et al. (2021) | 2015, AUD | TOTAL YEARLY COST | Overall | Lavender Suite: $620,858 | Hospital-based PC unit: $12,086,995 Residential high-care unit: $1,344,701 |
|  |  | Cost per person ($) | Overall | Lavender Suite: $10,178 | Hospital-based PC unit: $34,934 residential high-care unit: $44,823 |
|  |  | Cost per person per day | Overall | Lavender Suite: $242.33 | Hospital-based PC unit: $1,663.50 Residential high-care unit: $122.80 |
| Aldridge et al. (2023) | 2020, USD | Total health care costs at the end of life across all payers | Enrolment occurred last 3 days before death | 2,315 | 2,777 |
|  |  |  | Enrolment occurred last one week before death | 3,054 | 5,354 |
|  |  |  | Enrolment occurred last 2 weeks before death | 5,431 | 8,806 |
|  |  |  | Enrolment occurred last month before death | 10,578 | 15,059 |
|  |  |  | Enrolment occurred last 3 months before death | 32,644 | 36,061 |
|  |  |  | Enrolment occurred last 6 months before death | 59,180 | 60,697 |
